# Transcriptomic Atlas of Human Trabecular Meshwork Uncovers the Cellular Landscape and Provides Insights into Glaucoma Pathophysiology

**DOI:** 10.21203/rs.3.rs-9540265/v1

**Published:** 2026-06-22

**Authors:** GULAB ZODE, Prakadeeswari Gopalakrishnan, Pei Tan, Sarahi Arguello, Michael Nahmou, Zaid Ahmed, Linya Li, Yogapriya Sundaresan, Balasankara Reddy Kaipa, Lauren MacDonnell, Jinjing Jian, Mai-Linh Ton, Rui Chen, Xiangmin Xu

**Affiliations:** UCI; Robert M. Brunson Center for Translational Vision Research, University of California Irvine School of Medicine; University of California, Irvine; Robert M. Brunson Center for Translational Vision Research, School of Medicine, University of California,; Byers Eye Institute, Stanford University School of Medicine; Robert M. Brunson Center for Translational Vision Research, University of California Irvine School of Medicine; Robert M. Brunson Center for Translational Vision Research, University of California Irvine School of Medicine; Robert M. Brunson Center for Translational Vision Research, University of California Irvine School of Medicine; Robert M. Brunson Center for Translational Vision Research, University of California Irvine School of Medicine; Robert M. Brunson Center for Translational Vision Research, School of Medicine, University of California,; Robert M. Brunson Center for Translational Vision Research, University of California Irvine School of Medicine; Robert M. Brunson Center for Translational Vision Research, School of Medicine, University of California,; University of California Irvine; University of California Irvine

## Abstract

The trabecular meshwork (TM) is a specialized multicellular tissue that regulates aqueous humor outflow and intraocular pressure (IOP), and its dysfunction is a central driver of glaucoma. However, how cellular states and molecular mechanisms of TM cell populations are altered in human glaucoma remains poorly understood. Here, we present a comprehensive single-nucleus transcriptomic atlas of the human TM across normal and glaucomatous eyes. Analysis of 285,356 nuclei identified 17 distinct cell populations, including multiple TM structural subtypes, endothelial and neural-associated cells, and immune populations. Comparative analysis revealed widespread but cell-type–specific transcriptomic remodeling across TM populations in glaucoma, including dysregulation of metal ion homeostasis, inflammatory and interleukin signaling, disrupted calcium signaling, and activation of autophagy and mitophagy pathways. These changes were accompanied by altered extracellular matrix regulation, impaired endocytic processes, and enhanced stress-response and mechanosensitive signaling across TM populations. Notably, fibroblast- and myofibroblast-like TM populations exhibited transcriptomic signatures consistent with fibrotic remodeling and altered biomechanical responses, suggesting a potential role in increased outflow resistance. Together, these findings define a coordinated multicellular remodeling program linking proteostasis failure, mitochondrial dysfunction, inflammation, and fibrosis to TM failure in glaucoma, and highlight cell-type-specific therapeutic targets for restoring outflow and preventing vision loss.

## Introduction

Glaucomas are a complex group of chronic neurodegenerative disorders that cause irreversible vision loss, currently affecting an estimated 95 million individuals worldwide^1,2^. The global prevalence is projected to rise to 111.8 million by 2040^3,4^. Among these, Primary Open-Angle Glaucoma (POAG) is the most common form and a leading cause of irreversible blindness. It is characterized by progressive retinal ganglion cell (RGC) loss and optic nerve degeneration, with pathological changes occurring in both anterior and posterior segments of the eye, including the trabecular meshwork (TM), RGCs, lamina cribrosa, and optic disc. Elevated intraocular pressure (IOP) is the only known modifiable risk factor for disease onset and progression^1,4,5^. Elevated IOP in POAG arises primarily from increased resistance to aqueous humor (AH) outflow at the TM, the principal drainage tissue responsible for regulating IOP. Structural and functional alterations within the TM, particularly within the juxtacanalicular region, impair outflow facility, leading to sustained IOP elevation and downstream optic nerve damage in glaucoma. Consequently, lowering IOP remains the only proven therapeutic strategy for slowing glaucoma progression, and all currently approved treatments act by either reducing AH production or enhancing outflow through pharmacologic or surgical means. Despite the central role of TM dysfunction in glaucomatous IOP elevation, existing therapies do not directly target the underlying cellular and molecular mechanisms of the TM. Moreover, a substantial proportion of patients exhibit inadequate or diminishing responses to current IOP-lowering treatments, underscoring the limitations of symptom-based management. These observations highlight a critical unmet need to define the cellular composition, molecular programs, and disease-associated remodeling of the human TM. A deeper understanding of TM biology in health and glaucoma is essential for the development of mechanism-based therapies that restore outflow function and achieve durable IOP control.

The TM constitutes the principal site of resistance to AH outflow and is therefore central to IOP homeostasis. Functionally, the TM acts as a dynamic biological sieve and contractile filter that responds to ocular pulse, facilitating AH drainage into SC and subsequently the episcleral venous system^8^. The TM’s avascularity allows high permeability and effective filtration but confers limited self-repair capacity compared to vascularized tissues, rendering it particularly vulnerable to oxidative stress, mechanical strain, cellular debris overload, and accumulation of toxic byproducts from AH^6,7^. Lifetime exposure to these insults progressively reduces TM cellularity with aging and disease, leading to dysfunction, including impaired professional phagocytic activity, cytoskeletal alterations, and increased stiffness. Dysfunctional TM cells fail to maintain normal ECM remodeling, resulting in pathological deposition of collagen, fibronectin, and glycosaminoglycans, which increases outflow resistance and elevates IOP. In response to injury, TM cells can undergo a fibroblast-to-myofibroblast transition, characterized by ECM secretion and actomyosin contraction. While transient activation supports tissue repair, persistent activation drives pathological fibrosis, stiffening the TM and compromising its function^8,9^.

Anatomically, the TM is organized into three distinct regions; the inner uveoscleral meshwork (Uveal), the middle corneoscleral meshwork (CS), and the outer juxtacanalicular tissue (JCT), which is enriched in extracellular matrix (ECM) and located adjacent to the inner wall of Schlemm’s canal (SC). The Uveal and CS consist of collagen- and elastin-rich trabecular beams that provide structural support and filtration capacity. The JCT generates the majority of aqueous humor outflow resistance owing to its densely organized cells, enriched ECM, and narrow intercellular spaces at the inner wall of SC^6, 7^. TM cells originate from the neural crest and, despite exhibiting two distinct morphologies, share a common embryological origin^10^. These mesenchymal-derived cells are broadly classified into endothelial and fibroblastic phenotypes but uniquely display characteristics of endothelial, macrophage-like, fibroblastic, and smooth muscle-like phenotypes, supporting the TM’s primary functions. Endothelial TM cells, located in the uveoscleral and corneoscleral regions, maintain AH outflow, neutralize reactive oxygen species, and adopt macrophage-like roles contributing to filtration, phagocytosis, and immune regulation. Fibroblastic TM cells, enriched in the JCT and uveal, include fibroblasts responsible for ECM remodeling and tissue repair, alongside a subset exhibiting smooth muscle-like properties that regulate contractile tone and mechanotransduction^11^.

Single-cell RNA sequencing (scRNA-seq), single-nucleus RNA sequencing (snRNA-seq), and spatial transcriptomics enable high-resolution analysis of transcriptional variation and spatial organization across complex tissues^12–14^. Application of these approaches to the ocular anterior segment, including the TM, has begun to delineate TM cell populations^15–23^. However, most existing studies have focused on healthy human tissues or animal models, and considerable variability in reported TM cell subtypes and molecular signatures reflects an incomplete consensus on TM cellular classification.

Previous single-cell and spatial transcriptomic studies have defined the cellular composition, regional specialization, and transcriptional profiles of the human TM and adjacent aqueous humor outflow tissues. Cross-species and human atlases identified multiple TM and outflow-associated cell types and established a framework for cell-type classification and tissue organization, while comparisons between cultured and native TM revealed divergence in cellular markers and emphasized the importance of tissue-based profiling^20–23^. Spatial transcriptomic approaches in the TM further revealed region-specific gene expression patterns associated with ECM remodeling, cell adhesion, and integrin signaling^19,18^. Complementary studies in mouse and non-human primate models provided mechanistic insights into TM biology, highlighting roles of TM-SC signaling interactions, contractile gene expression patterns, and mitochondrial and stress-related pathways in IOP regulation and glaucoma pathogenesis, including structural and contractile alterations in TM^24–21^. Collectively, these studies establish a foundational understanding of TM cellular heterogeneity and function; however, how glaucomatous stress reshapes TM cellular composition and transcriptomic states in native human tissue remains insufficiently defined.

Existing TM transcriptomic analyses remain limited by small numbers of human samples, reliance on cultured cells, and a lack of direct, high-resolution comparisons between normal and glaucomatous human TM tissues. To more precisely define these disease-associated changes, we performed snRNA-seq of TM tissue from seven healthy and seven glaucomatous human donor eyes. This approach enabled unbiased profiling of native TM tissue while preserving transcriptomic signatures across diverse cell populations within the AH outflow pathway. Our analysis identified seventeen transcriptionally distinct TM cell populations and revealed widespread, cell-type-specific transcriptomic remodeling in glaucoma. Glaucomatous TM cells exhibited coordinated alterations in metal ion homeostasis, inflammatory and interleukin signaling, disrupted calcium signaling, and activation of autophagy and mitophagy pathways, together with changes in ECM regulation and cellular stress responses. These findings highlight convergent molecular mechanisms underlying TM dysfunction and impaired AH outflow in glaucoma.

## Results

### Single-nuclei transcriptomic map of the human TM in glaucoma and healthy individuals

**Figure 1:**
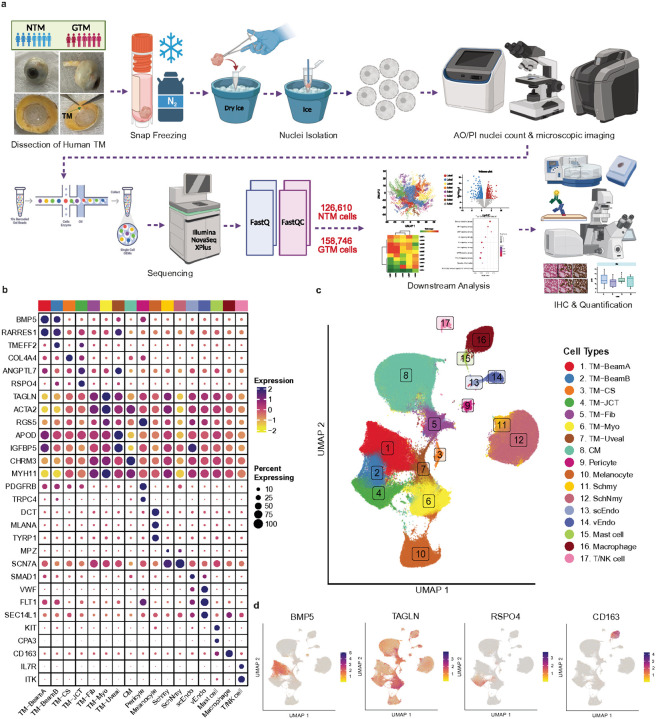
Single-nucleus transcriptomic profiling of normal and glaucomatous human TM. a) Schematic overview of the experimental workflow. Human TM tissue from NTM and GTM donor eyes was dissected, snap frozen, and processed for nuclei isolation. snRNA-seq libraries were generated and sequenced, followed by downstream computational analysis and validation by IHC. A total of 126,610 nuclei from NTM and 158,746 nuclei from GTM were analyzed. b) Dot plot showing expression of representative marker genes across identified cell populations. Dot size indicates the percentage of cells expressing each gene, and color intensity represents scaled expression levels. c) UMAP visualization of integrated single-nucleus transcriptomic data, showing 17 distinct cell populationsacross NTM and GTM samples. Identified populations include TM-BeamA (1; red), TM-BeamB (2; blue), TM-CS (3; orange), TM-JCT (4; green), TM-Fib (5; purple), TM-Myo (6; yellow), TM-Uveal (7; brown), CM (8; teal), pericytes (9; magenta), melanocytes (10; golden yellow), SchMy (11; mustard yellow), SchNmy (12; light pink), scEndo (13; light blue), vEndo (14; deep blue), mast cells (15; light green), macrophages (16; maroon), and T/NK cells (lilac pink) d) Feature plots showing expression of representative marker genes (*BMP5, TAGLN, RSPO4,* and *CD163*) across UMAP clusters, confirming cell-type-specific transcriptomic identities.

Several single-cell, single-nucleus, and spatial transcriptomic studies have begun to characterize the cellular composition and spatial organization of the human TM. However, how glaucomatous stress reshapes TM cellular states and transcriptional programs remains poorly understood. To define the cellular and molecular landscape of the human TM under healthy and glaucomatous conditions, we performed snRNA-seq on TM tissues isolated from seven NTM and GTM. Donor information is provided in Supplementary Table 1. A schematic overview of the experimental workflow from donor eye collection and TM dissection to snRNA-seq and downstream analysis is shown in Fig. 1a. Human cadaver eyes were obtained within 0–10 hours postmortem, and the TM region was carefully isolated using blunt dissection as previously described^22^. Representative Hematoxylin and eosin (H&E) images of the TM before and after dissection (Supplementary Fig. 1a-c) confirm clean isolation of TM tissue without contamination from adjacent ocular structures. After quality control filtering (Methods, Supplementary Fig. 2a-c, Supplementary Table 2), 285,356 high-quality nuclei corresponding to individual cells were retained for downstream analyses and used for cell-type clustering and cell proportion analysis, identifying 17 molecularly distinct cell types. Uniform Manifold Approximation and Projection (UMAP) visualization of the integrated dataset revealed 17 well-resolved clusters (Fig. 1c) defined by gene expression patterns corresponding to known TM and associated ocular cell populations. These included TM-juxtacanalicular tissue cells (TM-JCT), TM-corneoscleral meshwork cells (TM-CS), TM-uveoscleral meshwork cells (TM-Uveal), TM-beam cells (TM-BeamA and TM-BeamB), TM-fibroblast-like cells (TM-Fib), TM-myofibroblast-like cells (TM-Myo), ciliary muscle cells (CM), pericytes, melanocytes, Schlemm’s canal endothelial cells (scEndo), vascular endothelial cells (vEndo), Schwann myelinating (Schmy) and non-myelinating (SchNmy) cells, mast cells, macrophages, and T/NK cells (T cells and natural killer cells). These cell populations formed discrete clusters with clear cell-type-specific transcriptomic identities, indicating that cell identity is the primary driver of transcriptional heterogeneity within the integrated TM dataset. To examine condition-specific patterns, cells from NTM and GTM samples were visualized separately using UMAP projections (Supplementary Fig. 2f-g). Overlayed UMAP projections by individual sample (Supplementary Fig. 3a) and by condition (Supplementary Fig. 3b) demonstrated consistent clustering across donors and revealed no detectable batch effects. Cell counts for each cell type across individual donor samples are provided in Supplementary Table 3.

**Figure 2:**
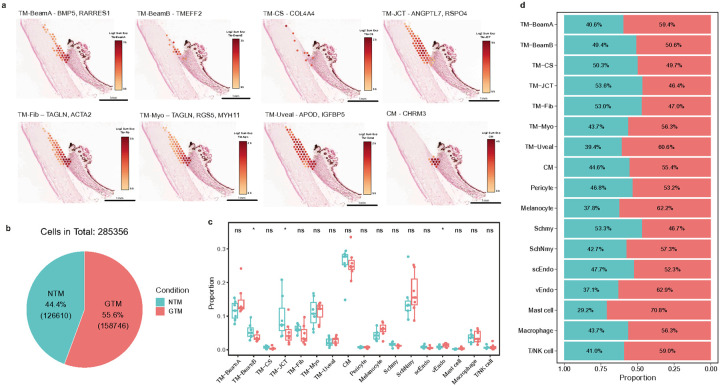
Cellular composition and spatial validation of TM cell populations in NTM and GTM a) Spatial transcriptomic validation of representative marker genes across TM regions. Visium spatial mappingshows log2-transformed summed expression of canonical marker genes for major TM subtypes, including TM-BeamA (BMP5, RARRES1), TM-BeamB (TMEFF2), TM-CS (COL4A4), TM-JCT (ANGPTL7, RSPO4), TM-Fib (TAGLN, ACTA2), TM-Myo (TAGLN, RGS5, MYH11), TM-Uveal (APOD, IGFBP5), and ciliary muscle cells (CM; CHRM3). Scale bars, 1 mm. b) Pie chart showing the total number and proportion of nuclei analyzed from normal (NTM; 126,610 cells, 44.4%) and glaucomatous (GTM; 158,746 cells, 55.6%) samples. c) Box plots showing the relative proportion of each identified cell population across NTM and GTM samples. Each point represents an individual donor sample; box plots indicate median and interquartile range. Statistical significance between conditions is indicated (Wilcoxon test; n of NTM=7, GTM=7; ns, not significant; *P < 0.05). d) Stacked bar plots showing the relative distribution of each cell population between NTM and GTM. Percentages indicate the proportion of cells contributed by each condition within each cell type.

To annotate and define the molecularly distinct transcriptomic clusters, we examined the expression of established marker genes using dot plot analysis (Fig. 1b). Marker genes used for cluster annotation are provided in Supplementary Table 4. The dot plot confirmed both the proportion of cells expressing each marker gene and their relative expression levels, thereby supporting the assigned cluster identities. To further visualize the distribution of marker genes within the transcriptional landscape, representative markers for each cluster were projected onto the UMAP embeddings. This analysis confirmed cluster-specific enrichment of canonical marker genes identified in the dot plot analysis, highlighting the transcriptomic diversity of cell populations within the human TM. Representative examples are shown in Fig. 1d, with additional markers presented in Supplementary Fig. 3c. To further resolve TM cellular diversity, we performed subclustering analysis of TM populations. TM subclusters were identified using the combined NTM and GTM dataset (Supplementary Fig. 4a) and subsequently visualized by condition (Supplementary Fig. 4b-c), with feature plots of marker genes confirming subpopulation identities (Supplementary Fig. 4d). While the overall cluster architecture was largely preserved between conditions, GTM samples exhibited shifts in subcluster representation relative to NTM. Specifically, TM-JCT, TM-BeamB, TM-Fib, and TM-CS subclusters were reduced in GTM, whereas TM-Myo, TM-Uveal, and TM-BeamA subclusters showed increased density in GTM. These annotations were further supported by Visium spatial transcriptomics of human NTM tissue, which confirmed the spatial localization of canonical marker genes used for cell clustering and annotation. Spatial mapping of representative marker genes for major TM cell subtypes and ciliary muscle cells is presented as log2-transformed summed expression values (Fig. 2a), whereas additional cell populations are shown to further support the annotation of remaining clusters (Supplementary Fig. 5). To assess the cellular distribution of genes previously implicated in glaucoma, we evaluated the expression of established glaucoma-associated genes across all identified cell populations. Dot plot analysis revealed variable cell-type-specific expression patterns of glaucoma-associated genes, including *PITX2, LMX1B, FOXC1, CYP1B1, MYOC, TMCO1, LRP12, ZFPM2, CNTNAP2, PLEKHA7, ST18, CDKN2B-AS1, GALC, COL11A1, CAV2, CAV1, MAF*, and *OPTN*, across TM structural, endothelial, neural, and immune populations (Supplementary Fig. 6). While several genes showed detectable expression in TM cell populations, others exhibited limited or minimal expression across the identified clusters, highlighting the diverse cellular contexts in which glaucoma-associated genes are represented within the TM microenvironment.

### Comparative analysis of TM cell-type composition between NTM and GTM

After defining the 17 TM cell populations, we examined the TM cell-type composition in 126,610 NTM cells and 158,746 GTM cells (Fig. 2b). Comparison of cell-type proportions across individual donors revealed consistent representation of major TM populations (Supplementary Fig. 2e), whereas condition-level comparisons between NTM and GTM revealed glaucoma-associated alterations in the relative abundance of specific TM cell types (Supplementary Fig. 2d). Comparison of mean cell-type proportions across donors revealed statistically significant alterations between NTM and GTM samples, with error bars reflecting donor-to-donor variability (Fig. 2c). Quantitative comparison of cell-type percentages further illustrated shifts in TM cellular composition in GTM relative to NTM (Fig. 2c). TM-JCT and TM-BeamB cells were significantly reduced in GTM, while TM-CS cells also showed a modest decrease. In contrast, TM-BeamA and TM-Uveal populations were increased, and vascular endothelial (vEndo) cells showed an expansion. TM-Fib cells were reduced, whereas TM-Myo cells were relatively increased. Immune populations, including macrophages and T/NK cells, showed higher proportions in GTM samples. Additionally, Schwann cell populations exhibited reciprocal changes, with Schmy cells decreasing and SchNmy cells increasing. Together, these results indicate condition-associated shifts and remodeling of TM cellular composition in GTM.

### Cell-type-specific differential gene expression and pathway enrichment in GTM

To investigate transcriptional changes underlying the observed shifts in TM cell populations, we performed cell-type-specific differential expression (DE) analysis between GTM and NTM samples. Volcano plot analysis of individual cell types revealed distinct sets of upregulated and downregulated genes, with log_2_ fold change plotted against −log_10_ adjusted p-value, indicating widespread transcriptional alterations in glaucoma.

### TM-JCT cells

**Figure 3:**
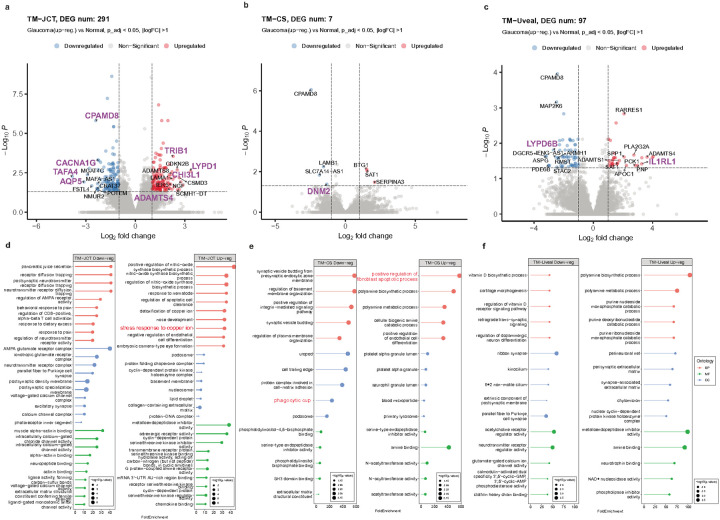
DEGs and pathway enrichment across TM cell populations in glaucoma. Volcano plots representing DEGs across TM cell populations comparing GTM versus NTM conditions. Volcano plots for TM-JCT (a, n = 291 DEGs), TM-CS (b, n = 7 DEGs), TM-Uveal (c, n = 97 DEGs) show genes plotted as log_2_ fold change versus −log_10_ P value. Upregulated genes are shown in orange, downregulated genes in blue, and non-significant genes are shown in gray, with selected key genes annotated. Thresholds were set at adjusted P < 0.05 and log_2_ fold change > 1. Corresponding GO enrichment analyses are shown for TM-JCT (d), TM-CS (e), and TM-Uveal (f), where lollipop plots display enriched pathways ranked by fold enrichment. Downregulated pathways are shown on the left and upregulated pathways on the right. Dot size represents gene count, and color indicates statistical significance (−log_10_ adjusted P value). Ontology categories are indicated as BP (pink), CC (blue), and MF (green).

A total of 291 genes were differentially expressed in GTM, as illustrated in the volcano plot (Fig. 3a, Supplementary Table 5). DE expression analysis identified a distinct transcriptional profile in the TM-JCT cluster. The most highly upregulated genes included *LYPD1*, *CHI3L1*, *TRIB1*, *ADAMTS4*, ADAMTS8, *LAMA1*, *CDKN2B*, *IER5*, *NGF*, *CSMD3*, and *SCMH1-DT*. In contrast, the most highly downregulated genes included *CPAMD8*, *CACNA1G*, *AQP5*, *TAFA4*, *MGAT4C*, *MAFA-AS1*, *NMUR2*, *FSTL4*, and CRAT37. These differentially expressed genes (DEGs) were subsequently analyzed to identify enriched pathways and biological processes in the TM-JCT cells. Pathway enrichment analysis revealed upregulation of nitric oxide synthase biosynthesis and its positive regulation, increased collagen-containing ECM, podosome assembly, stress and detoxification responses to copper ions, enhanced regulation of apoptotic cell clearance, enrichment of metalloendopeptidase inhibitor activity, and downregulation of voltage-gated calcium channels (Fig. 3d, Supplementary Table 6). The downregulation of calcium channels may reduce intracellular calcium signaling, potentially affecting TM contractility and ECM dynamics. These coordinated changes suggest elevated NO signaling with ECM remodeling, restricted ECM proteolysis, adaptation to metal ion stress, and maintenance of tissue homeostasis, which may collectively influence aqueous humor outflow and tissue integrity.

### TM-CS cells

Seven genes were differentially expressed in GTM, as visualized in the volcano plot (Fig. 3b; Supplementary Table 5), highlighting distinct molecular features of the corneoscleral TM region. The most highly upregulated genes included *BTG1*, *SAT1*, and *SERPINA3*. In contrast, the most highly downregulated genes included *DNM2*, *LAMB1*, *SLC7A14 - AS1*, and *CPAMD8*. The identified DEGs were subjected to pathway and functional enrichment analyses to characterize the biological processes associated with TM-CS cells. Pathway enrichment analysis revealed downregulation of phagocytic cup formation, podosome assembly, cell trailing edge dynamics, protein complexes involved in cell-matrix adhesion, ECM structural constituents, and synaptic vesicle budding processes. Upregulated pathways included positive regulation of fibroblast apoptotic processes, polyamine biosynthesis and metabolism, primary lysosome biogenesis, and N-acetyltransferase activity (Fig. 3e, Supplementary Table 6). These coordinated changes suggest reduced TM cell motility, phagocytosis, and ECM remodeling, alongside increased cellular stress responses and lysosomal activity, which may collectively contribute to altered tissue homeostasis in the corneoscleral TM.

### TM-Uveal cells

In GTM, 97 genes were found to be differentially expressed (Fig. 3c; Supplementary Table 5), revealing a molecular profile unique to the uveoscleral TM region. The most highly upregulated genes included *ADAMTS1*, ADAMTS4, *SAT1*, *PLA2G2A*, *SPP1*, *PCK1*, *IL1RL1*, *APOC1*, and *PNP*. In contrast, the most highly downregulated genes included *CPAMD8*, *MAP2K6*, *LYPD6B*, *ASPG*, *PDE6B*, *IFNG-AS1*, *ARMH1*, *DGCR5*, and *STAC2*. Pathway and functional enrichment analyses were performed on the identified DEGs to define the biological processes associated with TM-Uveal cells. These analyses revealed downregulation of kinocilium formation, 9 + 2 non-motile cilium function, glutamate-gated calcium ion channel activity, vitamin D biosynthesis and receptor signaling, retrograde trans-synaptic signaling, extrinsic components of the postsynaptic membrane, neurotransmitter receptor activity, and parallel fiber to Purkinje cell synapse-related processes. Upregulated pathways included polyamine biosynthesis and metabolism, chylomicron formation, perisynaptic and synapse-associated ECM components, metalloendopeptidase inhibitor activity, amine binding, neurotrophin binding, NAD^+^ nucleosidase activity, and phospholipase inhibitor activity (Fig. 3f, Supplementary Table 6).

### TM-BeamA cells

**Figure 4:**
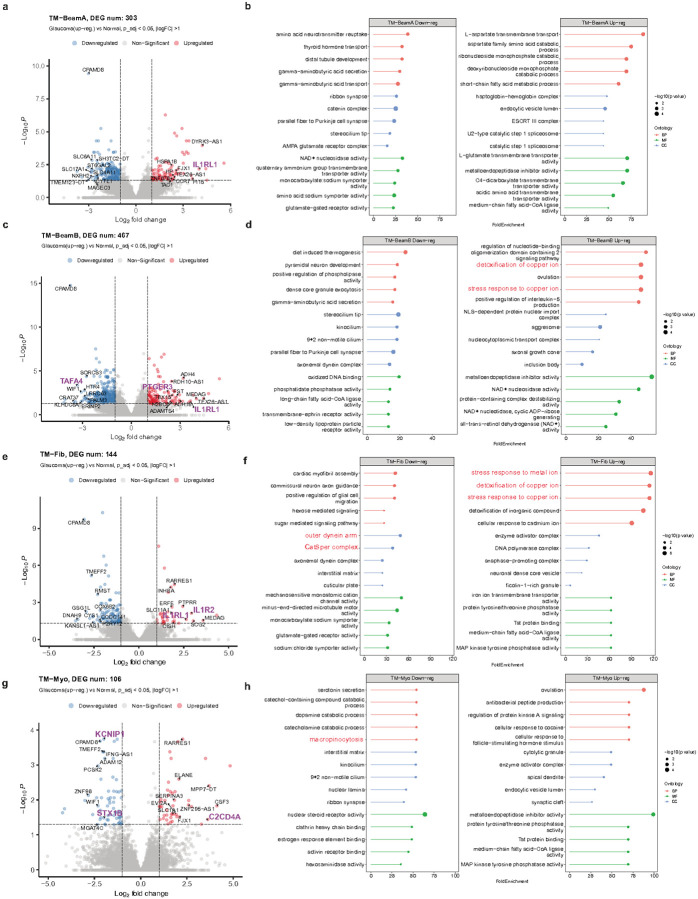
DEGs and pathway enrichment across TM subpopulations in glaucoma. Volcano plots representing DEGs across TM subpopulations comparing GTM versus NTM conditions. Volcano plots for TM-BeamA (a, n = 303 DEGs), TM-BeamB (c, n = 467 DEGs), TM-Fib, (e, n = 144 DEGs) and TM-Myo (g, n = 106 DEGs), show genes plotted as log_2_ fold change versus −log_10_ P value. Upregulated genes are shown in orange-red, downregulated genes in blue, and non-significant genes are shown in gray, with selected key genes annotated. Thresholds were set at adjusted P < 0.05 and log_2_ fold change > 1. Corresponding GO enrichment analyses are shown for TM-BeamA (b), TM-BeamB (d), TM-Fib (f) and TM-Myo (h), where lollipop plots display enriched pathways ranked by fold enrichment. Downregulated pathways are shown on the left and upregulated pathways on the right. Dot size represents gene count, and color indicates statistical significance −log_10_ adjusted P value). Ontology categories are indicated as BP, CC, and MF.

Analysis of GTM identified 303 DEGs, defining a unique molecular signature of BeamA cells within the TM (Fig. 4a; Supplementary Table 5). The most highly upregulated genes included *IL1RL1*, *DYRK3-AS1*, *HSPA1B*, *FJX1*, HP, *ZNF878*, *TAC1*, *CCR7*, *TEX26-AS1*, and *PI15*. In contrast, the most highly downregulated genes included *CPAMD8*, *SLC4A11*, *SLC6A11*, *SLC17A1*, *NXPH2*, *TMEM123-DT*, *MAGEC3*, *CYTL1*, *ST6GAL2*, and *SH3TC2-DT*. Pathway and functional enrichment analyses of these DEGs were conducted to define the biological processes associated with TM-BeamA cells (Fig. 4b; Supplementary Table 6). Downregulated pathways included catenin complex, stereocilium tip, NAD^+^ nucleosidase activity, glutamate-gated receptor activity, ribbon synapse, parallel fiber to Purkinje cell synapse, amino acid neurotransmitter reuptake, and AMPA glutamate receptor complex. Upregulated pathways included endocytic vesicle lumen, ESCRT-III complex, catalytic step 1 spliceosome, L-glutamate and L-aspartate transmembrane transporter activity, and metallopeptidase inhibitor activity.

### TM-BeamB cells

In GTM, 467 genes were differentially expressed in TM-BeamB cells, uncovering a specialized molecular profile of this TM subpopulation (Fig. 4c; Supplementary Table 5). The most highly upregulated genes included *PTGER3*, *ADH4*, *ADH1B*, *RDH10 - AS1*, *TBX15*, *MEDAG*, *FST, H2BC8*, *TEX26-AS1*, and *ADAMTS4*. In contrast, the most highly downregulated genes included *SORCS3*, *WIF1*, *HTR4*, *CRAT37*, *BRINP2*, *PALM3*, *KLHDC8A*, *CPAMD8*, and *TAFA4*. Enrichment analyses of these DEGs highlighted the key molecular pathways and biological processes associated with TM-BeamB cells (Fig. 4d; Supplementary Table 6). Downregulated pathways included pyramidal neuron development, positive regulation of phospholipase activity, dense core granule exocytosis, stereocilium tip, kinocilium, 9 + 2 non-motile cilium, parallel fiber to Purkinje cell synapse, axonemal dynein complex, phosphatidate phosphatase activity, transmembrane-ephrin receptor activity, and low-density lipoprotein particle receptor activity. Upregulated pathways included detoxification and stress response to copper ions, positive regulation of interleukin-5 production, NSL-dependent protein nuclear import complex, aggresome formation, nucleocytoplasmic transport complex, inclusion body formation, metalloendopeptidase inhibitor activity, NAD^+^ nucleosidase activity, and protein-containing complex destabilizing activity

#### TM-Fib cells:

In GTM, significant transcriptional differences were observed for 144 genes in TM-Fib cells, as illustrated in the volcano plot (Fig. 4e; Supplementary Table 5). The most highly downregulated genes included *CPAMD8*, *TMEFF2*, *RMST*, *GSG1L*, *CYS1*, *DNAH9*, *KANSL1-AS1*, *P2RY12*, *COX6B2*, and *CCDC141*. In contrast, the upregulated genes included *RARRES1*, *MEDAG*, *IL1RL1*, *IL1R2*, *INHBA*, *PTPRR*, *ERFE*, *SLC11A1*, SCG2, and *CISH*. Pathway enrichment analysis was subsequently performed on these differentially expressed genes to define the biological processes in TM-Fib cells (Fig. 4f; Supplementary Table 6). Upregulated pathways included stress responses to metal and copper ions, detoxification of copper ions, cellular response to cadmium ions, iron ion transmembrane transporter activity, MAP kinase tyrosine phosphatase activity, protein tyrosine/threonine phosphatase activity, anaphase-promoting complex, and DNA polymerase complex. In contrast, downregulated pathways were enriched for outer dynein arm, CatSper complex, axonemal dynein complex, mechanosensitive monoatomic cation channel activity, minus-end-directed microtubule motor activity, monocarboxylate: sodium symporter activity, glutamate-gated receptor activity, and sodium: chloride symporter activity.

### TM-Myo cells

A total of 106 genes exhibited differential expression in GTM in TM-Myo cells (Fig. 4g; Supplementary Table 3). Downregulated genes included *CPAMD8*, *TMEFF2*, *WIF1*, *STX1B*, *KCNIP1*, *ZNF98*, *ADAM12*, *MGAT4C*. Upregulated genes included *RARRES1*, *SERPINA3*, *ELANE*, *MPP7-DT*, *EVI2A*, *SLC1A1*, *ZNF295-AS1*, *FJX1*, *C2CD4A*. These differential transcriptomic gene alteration profiles in TM-Myo cells enabled the identification of enriched pathways in GTM cells (Fig. 4h; Supplementary Table 6). Downregulated pathways included micropinocytosis, kinocilium, 9 + 2 non-motile cilium, and ribbon synapses. Upregulated pathways included MAP kinase tyrosine phosphatase activity, protein tyrosine/threonine phosphatase activity, metalloendopeptidase inhibitor activity, clathrin heavy chain binding, endocytic vesicle lumen, and cytolytic granule. Altogether, comprehensive analyses of DEGs and pathway enrichment across the other distinct TM cell clusters identified in this study are provided in the Supplementary Information (Supplementary Figs. 7a-g, 8a-f, 9a-f). Building on these analyses, we next examined the molecular signatures that characterize human GTM.

### Molecular signatures of GTM

**Figure 5:**
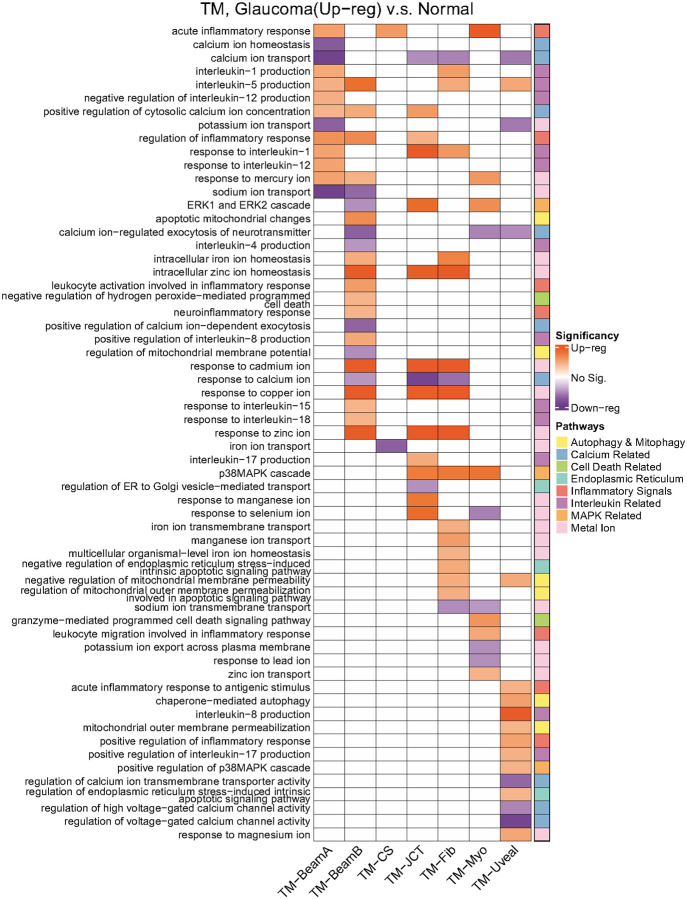
Summary heatmap of pathway enrichment across TM cell populations in glaucoma. Heatmap summarizing differential pathway enrichment between GTM and NTM samples across TM cell populations (TM-BeamA, TM-BeamB, TM-CS, TM-JCT, TM-Fib, TM-Myo, and TM-Uveal). Rows represent significantly enriched biological pathways, and columns represent TM cell types. Color indicates enrichment relative to normal samples, with upregulated pathways (increased in GTM) shown in orange, downregulated pathways (decreased in GTM) in purple, and non-significant pathways in white. Pathways are grouped into major functional categories and color-coded as follows: metal ion homeostasis (light pink), interleukin-related pathways (violet), calcium signaling (blue), inflammatory pathways (red), autophagy and mitophagy (yellow), endoplasmic reticulum-related pathways (teal), MAPK signaling (mustard), and cell death-related pathways (green).

To define the molecular landscape of GTM, we performed pathway enrichment analysis across transcriptionally distinct TM cell clusters. Heatmap visualization of significantly up- and downregulated pathways (Fig. 5) revealed pronounced cluster-specific enrichment patterns, indicating distinct molecular signatures across GTM cell populations. Pathway categorization identified key biological processes with distinct distribution patterns, with metal ion-related pathways (light pink; 19 pathways) being most prevalent, followed by interleukin-related pathways (violet; 12), calcium signaling (blue; 8), inflammatory pathways (saffron red; 7), and autophagy/mitophagy (yellow; 6). Endoplasmic reticulum-related pathways (teal; 3), MAPK-related pathways (mustard; 2), and cell death-related pathways (green; 2) were less frequent. Based on enrichment frequency and statistical significance, metal ion regulation, interleukin signaling, calcium signaling, inflammatory pathways, and autophagy/mitophagy were prioritized as key mechanisms underlying GTM heterogeneity.

### Metal ions

**Figure 6:**
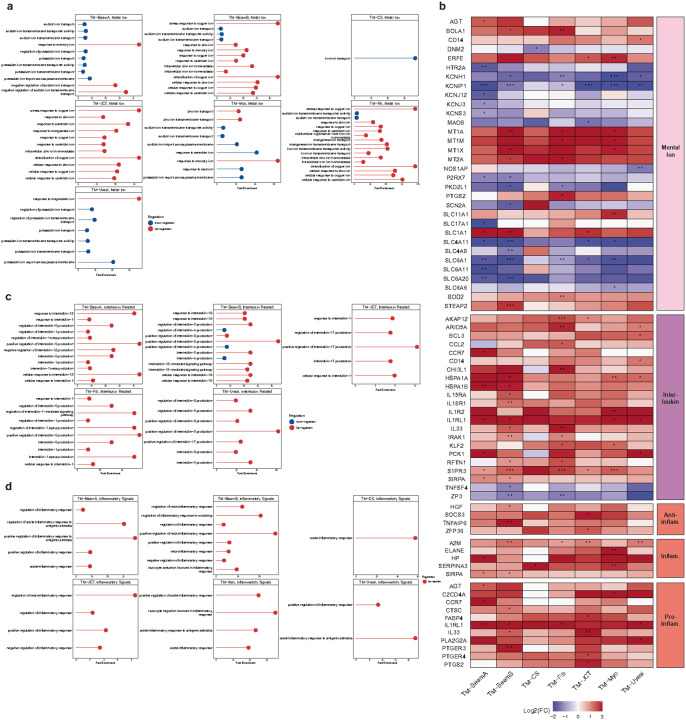
Cell-type-specific interleukin signaling, inflammatory responses, and metal ion regulation in GTM. a) GO enrichment analysis of metal ion-related pathways across TM cell populations (TM-BeamA, TM-BeamB,TM-CS, TM-JCT, TM-Fib, TM-Myo, and TM-Uveal). b) Heatmap showing DE (log_2_ fold change) of representative genes associated with metal ion homeostasis, interleukin signaling, and inflammatory pathways across TM cell populations. Color scale represents relative expression fold of changes (GTM vs NTM), with red indicating upregulation and blue indicating downregulation. Functional categories are indicated at right, including metal ion-related, interleukin-related, anti-inflammatory, inflammatory, and pro-inflammatory genes. Pathways are grouped and color-coded as follows: metal ion homeostasis (light pink), interleukin-related pathways (violet), and inflammatory pathways (red). The fold-of change and pvalue are derivative from DE analyses. c & d) GO enrichment analysis of interleukin-related (c) and inflammatory signaling (d) pathways across TM cell populations. Pathways are shown as fold enrichment, with upregulated pathways (increased in GTM) indicated in red and downregulated pathways (decreased in GTM) in blue.

Metal ions-related pathways were the most prominent features of the pathway enrichment heatmap (Fig. 5), representing a major molecular signature of the GTM. To delineate cell-type contributions, we examined cluster-specific metal ion-associated pathways and DEGs to identify potential mechanisms relevant to GTM (Fig. 6a & 6b). TM-JCT cells showed broad upregulation of metal stress and detoxification pathways, including responses to copper, zinc, selenium, manganese, and cadmium ions, as well as intracellular zinc homeostasis and copper detoxification, indicating heightened metal-induced stress. TM-Uveal cells exhibited upregulation of magnesium ion-responsive pathways with concurrent downregulation of potassium ion transport. TM-Fib cells showed increased metal detoxification pathways (copper, zinc, cadmium, iron) alongside enhanced iron transport and reduced sodium transport. TM-Myo cells displayed upregulation of zinc transport and mercury response pathways, with downregulation of sodium transport, selenium and lead responses, and potassium export (Fig. 6a).

Metallothionein (*MT*) family genes were prominently upregulated across TM populations, with *MT1M*, *MT2A*, *MT1A*, and *MT1X* showing strongest expression in TM-BeamB, TM-JCT, and TM-Fib cells. Consistent with oxidative stress, *SOD2* was significantly upregulated in TM-JCT cells, while *STEAP2* expression was elevated in TM-BeamB cells. Subtype-specific DE were observed across TM populations. *P2RX7* expression was reduced in TM-BeamA and TM-BeamB cells, while *PKD2L* was significantly decreased in TM-BeamB and TM-JCT cells. In contrast, *PTGS2* was significantly upregulated in TM-JCT and broadly elevated across TM populations. *AGT* expression was markedly increased in TM-Beam cells and moderately elevated in other TM subtypes, with minimal expression in TM-CS cells. Additional changes included upregulation of *BOLA1* in TM-JCT and TM-Beam cells and reduced *HTR2A* expression in TM-BeamA, with moderate decreases in TM-BeamB and TM-Uveal cells. *NOS1AP* was significantly reduced in TM-Uveal cells (Fig. 6b).

Solute carrier (SLC) transporter genes showed widespread transcriptional remodeling. MA-plot analysis (Supplementary Fig. 10a) confirmed differential regulation of SLC family genes involved in zinc, copper, iron, lead, sodium, and manganese transport. Pathway enrichment (Supplementary Fig. 10b) highlighted metal ion transport, homeostasis, and cation channel regulation. *SLC11A1* was upregulated in TM-Fib cells, while *SLC1A1* was increased in TM-BeamA and TM-JCT cells. In contrast, *SLC17A1, SLC4A11, SLC6A11, SLC4A8, SLC6A1, SLC6A20*, and *SLC6A6* were downregulated across TM subtypes. Consistent with disrupted ion transport, potassium channel genes were broadly downregulated. MA-plot analysis (Supplementary Fig. 11a) showed *KCNH1* in TM-BeamB, TM-JCT, TM-Fib, and TM-Uveal cells, while *KCNIP1* was decreased across all TM populations. Additional potassium channel genes (*KCNJ3*, *KCNJ12*, *KCNS3*) were selectively reduced in TM-BeamA cells, with pathway analysis confirming the suppression of potassium channel activity (Supplementary Fig. 11b). Collectively, these data indicate pronounced cell-type–specific alterations in metal ion homeostasis, transport, and detoxification, highlighting specialized metal-responsive molecular profiles that may contribute to GTM-associated cellular stress and dysfunction.

### Interleukin-related pathway

Interleukin-related pathways represented a major category of enrichment in GTM, highlighting their central role in TM cell heterogeneity. (Fig. 5). TM-JCT cells showed strong enrichment of pathways associated with cellular responses to IL-1 and the production, regulation, and negative regulation of IL-17 (Fig. 6c). TM-Uveal cells were enriched for pathways involved in the production and regulation of IL-5 and IL-8, including positive regulation of IL-5, IL-8, and IL-17 production. TM-BeamA cells exhibited enrichment of pathways related to the production of IL-1, IL-5, and IL-1β, as well as cellular responses to IL-1 and IL-12, with positive regulation of IL-5 and negative regulation of IL-12. TM-BeamB cells were enriched for pathways associated with cellular responses to IL-15 and IL-18, along with reduced IL-4 production and increased positive regulation of IL-5 and IL-8.

TM-Fib cells showed enrichment of IL-1-related pathways, including cellular responses to IL-1, regulation of IL-1-mediated signaling, and production of IL-1, IL-5, and IL-1α (Fig. 6c). Consistent with these pathway-level observations, genes associated with immune signaling and inflammatory regulation displayed region-specific expression across TM compartments (Fig. 6b). Consistent with these patterns, immune- and stress-related genes displayed region-specific expression. *AKAP12* was upregulated in TM-JCT and TM-Myo cells, *ARID5A* in TM-JCT and TM-Uveal cells, and *BCL3* in TM-Uveal cells. Stress-response genes *HSPA1A* and *HSPA1B* were strongly induced in beam-associated regions, with *HSPA1A* elevated in TM-BeamB, TM-Fib, and TM-Uveal cells and *HSPA1B* in TM-BeamA and TM-BeamB cells.

Interleukin-associated genes showed distinct expression patterns. *IL15RA* and *IL18R1* were upregulated in TM-BeamB cells, while *IL1RL1* was elevated in TM-BeamA, TM-BeamB, TM-Fib, and TM-Uveal cells. *IL1R2* was enriched in TM-BeamA, TM-CS, TM-Fib, and TM-Uveal cells, and *IL33* was elevated in TM-JCT and TM-BeamB cells. *IRAK1* was upregulated in TM-BeamB and TM-JCT cells, while *KLF2* was enriched in TM-JCT and TM-Fib cells. *CCL2* was upregulated in TM-JCT and TM-BeamA cells, and *CCR7* in TM-BeamA cells. *CD14* was enriched in TM-Uveal cells, and *CHI3L1* in TM-JCT cells. *PCK1* was upregulated in TM-BeamA and TM-Uveal cells, while *RFTN1* was increased in TM-BeamB and TM-JCT cells. *S1PR3* showed strong enrichment across TM-BeamA, TM-BeamB, TM-JCT, and TM-Fib cells, and *SIRPA* was upregulated in TM-BeamA and TM-BeamB cells. In contrast, TNFSF4 was downregulated in TM-BeamB cells, and *ZP3* in TM-BeamB and TM-JCT cells. Collectively, these findings highlight pronounced regional heterogeneity in interleukin-associated gene expression across TM compartments, reflecting differential regulation of immune and inflammatory processes.

### Inflammatory response pathway

In addition to interleukin-related pathways, inflammatory response pathways were enriched across TM cell populations, indicating heightened inflammatory activation alongside regulatory responses in GTM (Fig. 5). Consistent with this, inflammatory pathways were broadly upregulated across TM clusters (Fig. 6d). Our dataset revealed concurrent activation of both pro-inflammatory and anti-inflammatory gene programs across TM populations (Fig. 6b). Anti-inflammatory genes *HGF* and *TNFAIP6* were enriched in TM-BeamB cells, while *SOCS3* and *ZFP36* were elevated in TM-JCT cells. In contrast, pro-inflammatory genes showed subtype-specific expression, with *AGT* and *CCR7* upregulated in TM-BeamA cells, *CTSC* and *PTGER3* in TM-BeamB cells, *C2CD4A* in TM-Myo cells, and *FABP4* in TM-JCT cells. Additional genes included *A2M* in TM-BeamB, TM-JCT, TM-Fib, and TM-Uveal cells, *ELANE* in TM-Myo cells, *HP* in TM-BeamA cells, and *SERPINA3* in TM-CS and TM-Myo cells. These analyses highlight the coexistence of pro-inflammatory activation and compensatory anti-inflammatory responses across TM compartments, indicating a tightly regulated inflammatory environment in GTM.

### Calcium ion-related

**Figure 7:**
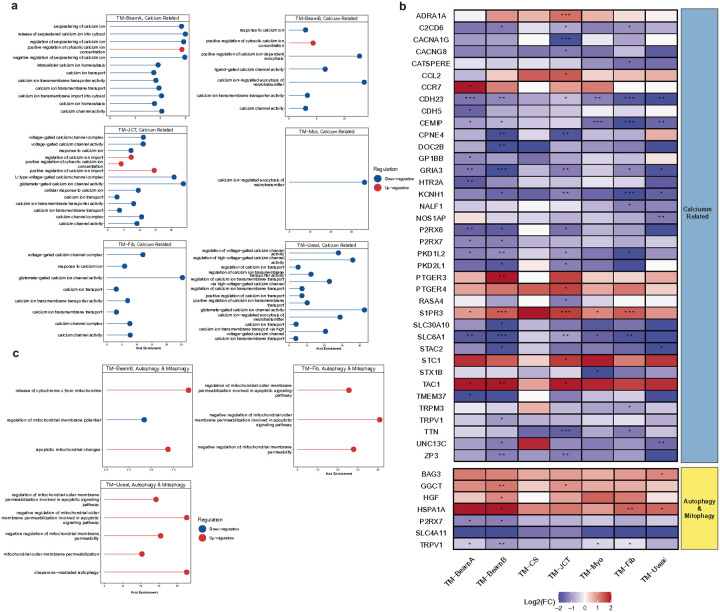
Cell-type-specific calcium signaling and autophagy/mitophagy pathways in GTM. a) GO enrichment analysis of calcium-related pathways across TM cell populations (TM-BeamA, TM-BeamB,TM-CS, TM-JCT, TM-Fib, TM-Myo, and TM-Uveal). b) Heatmap showing DE (log_2_ fold change) of representative genes associated with calcium signaling andautophagy/mitophagy across TM cell populations. Color scale represents relative expression changes (GTM vs NTM), with red indicating upregulation and blue indicating downregulation. Functional groupings are indicated at right, including calcium-related (blue) and autophagy/mitophagy (yellow) gene sets. c) GO enrichment analysis of autophagy- and mitophagy-related pathways across TM cell populations. Pathways are shown as fold enrichment, with upregulated pathways (increased in GTM) indicated in red and downregulated pathways (decreased in GTM) in blue.

Calcium ion-related pathways represented a major category of enrichment in GTM (Fig. 5), with widespread downregulation across TM cell populations, indicating dysregulated calcium signaling as a key contributor to TM heterogeneity. Across TM-JCT, TM-Uveal, TM-BeamA, TM-BeamB, and TM-Fib cells, pathways associated with voltage- and ligand-gated calcium channel activity, calcium transport, transmembrane flux, and cellular responses to calcium were broadly downregulated (Fig. 7a). In addition, calcium-dependent exocytosis pathways were consistently reduced in TM-Uveal, TM-BeamB, and TM-Myo cells. Despite this overall suppression, selective upregulation of pathways regulating calcium ion import and cytosolic calcium levels were observed in TM-JCT and TM-BeamA cells, indicating compensatory regulation of intracellular calcium homeostasis. Furthermore, TM-BeamA cells showed additional downregulation of calcium sequestration and intracellular calcium homeostasis pathways, while TM-Myo cells predominantly exhibited reduced calcium-regulated exocytosis (Fig. 7a).

Consistent with pathway enrichment results, genes associated with calcium signaling, ion transport, and membrane trafficking showed compartment-specific differential expression across TM populations (Fig. 7b). *ADRA1A* and *CCL2* were upregulated in TM-JCT cells, while *CCR7* was enriched in TM-BeamA cells. In contrast, calcium channel–associated genes, including *CACNA1G*, *CACNG8*, *CATSPERE*, and *C2CD6*, were broadly downregulated, with *CACNA1G* and *CACNG8* reduced in TM-JCT cells, *CATSPERE* in TM-Fib cells, and *C2CD6* across TM-BeamB, TM-JCT, and TM-Fib cells. Genes involved in calcium-dependent membrane signaling and vesicle trafficking, including *CEMIP*, *CPNE4*, and *DOC2B*, were also consistently downregulated across multiple TM compartments. Similarly, several ion channel genes, including *GRIA3*, *P2RX6*, *P2RX7*, *KCNH1*, *PKD1L2*, and *PKD2L1*, showed widespread downregulation, indicating impaired ion channel function. Structural and adhesion-related genes such as *CDH23*, *CDH5*, *GP1BB*, and *HTR2A* were also reduced. In contrast, select signaling-related genes showed cell-type–specific upregulation. *PTGER3* was increased in TM-BeamB cells, *PTGER4* and *STC1* in TM-JCT cells, and *S1PR3* across TM-BeamA, TM-JCT, TM-Myo, and TM-Fib cells. The neuropeptide gene *TAC1* showed strong enrichment in TM-BeamA, TM-BeamB, and TM-JCT cells. Conversely, vesicle-associated genes, including *STX1B*, *STAC2*, and *UNC13C*, were downregulated across TM populations. Additional genes, including *SLC6A1*, *SLC30A10*, *TMEM37*, *TRPM3*, *TRPV1*, *TTN*, and *ZP3*, also exhibited predominant downregulation across TM compartments. These data highlight widespread alterations in ion channel function, vesicle trafficking, and calcium-dependent signaling, consistent with disrupted calcium homeostasis across TM populations.

### Autophagy and mitophagy

Autophagy and mitophagy pathways were significantly enriched in GTM (Fig. 5), highlighting key mechanisms contributing to TM cell heterogeneity. TM-BeamB cells showed enrichment of mitochondrial apoptosis–related pathways, including cytochrome c release and apoptotic mitochondrial changes, alongside downregulation of mitochondrial membrane potential regulation (Fig. 7c). TM-Fib cells exhibited strong enrichment of mitochondrial outer membrane permeabilization and its regulation, while TM-Uveal cells showed enrichment of mitophagy-related processes, including mitochondrial outer membrane permeabilization and chaperone-mediated autophagy. Together, these findings indicate activation of mitochondrial quality control and autophagy pathways across specific TM populations in GTM. Consistent with these pathway-level changes, several autophagy- and mitophagy-associated genes displayed compartment-specific differential expression across TM populations (Fig. 7b). *BAG3* showed increased expression across multiple TM compartments, with strong enrichment in TM-Uveal cells. *GGCT* was elevated in TM-BeamB and TM-JCT cells, while *HGF* showed increased expression in TM-BeamB cells. The stress-response gene *HSPA1A* was strongly upregulated across TM-BeamB, TM-Fib, and TM-Uveal cells. In contrast, ion channel–related genes *P2RX7* and *TRPV1* were downregulated in TM-BeamA/TM-BeamB and TM-BeamB cells, respectively, and the transporter gene *SLC4A11* showed pronounced downregulation across TM-BeamA, TM-BeamB, TM-Myo, and TM-Fib cells. These findings indicate coordinated alterations in autophagy- and mitophagy-related gene expression across TM populations in GTM.

### Mitogen-activated protein kinase (MAPK)-related

**Figure 8:**
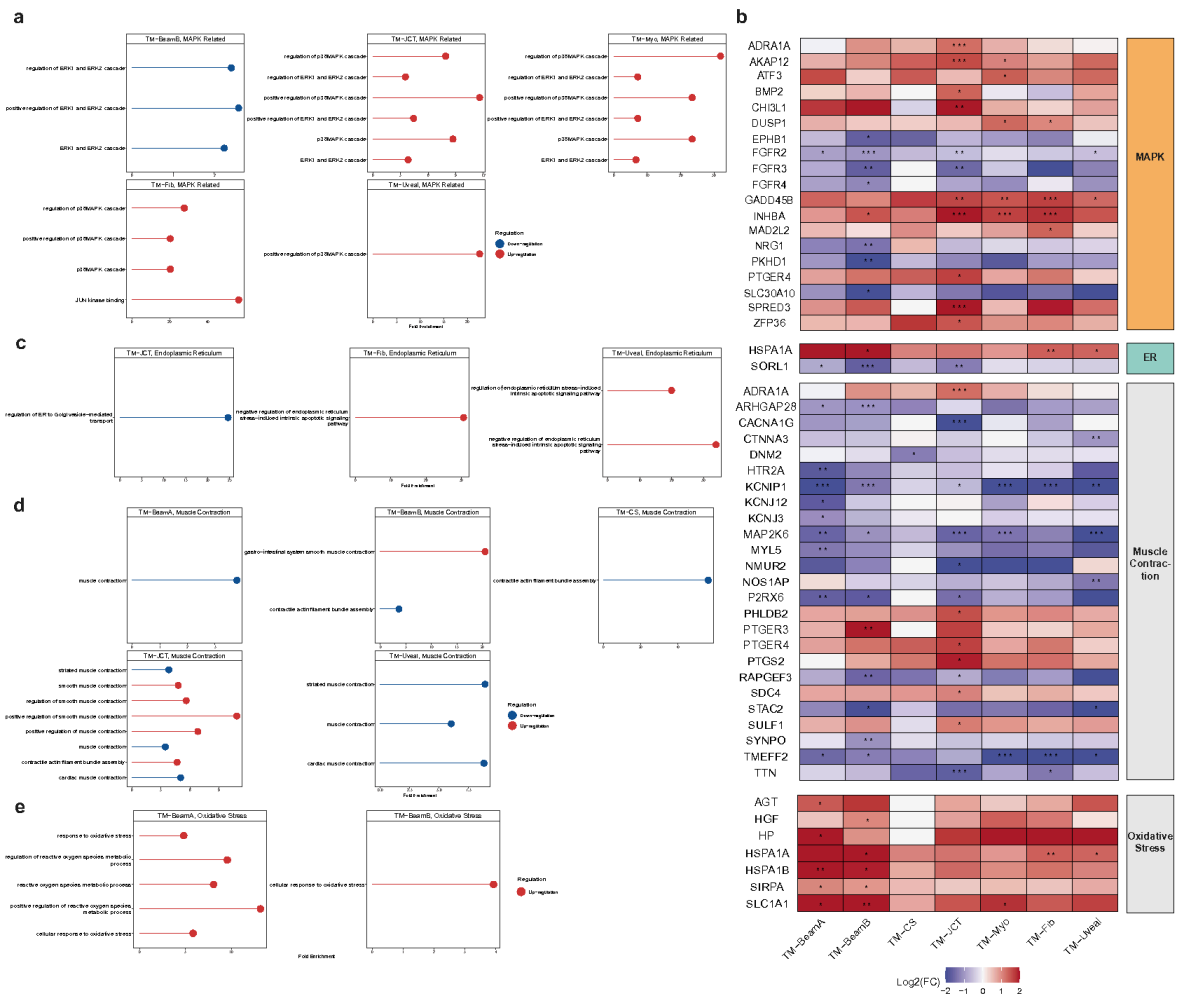
Cell-type-specific MAPK signaling, ER stress, muscle contraction, and oxidative stress pathways in GTM. a) GO enrichment analysis of MAPK-related pathways across TM cell populations. b) Heatmap showing differential expression (log_2_ fold change) of representative genes associated with MAPKsignaling, ER stress, muscle contraction, and oxidative stress across TM cell populations. Color scale represents relative expression changes (GTM vs NTM), with red indicating upregulation and blue indicating downregulation. Functional groupings are indicated at right, including MAPK (orange), ER-related (teal), muscle contraction (grey), and oxidative stress (red) gene sets. c-e) GO enrichment analysis of ER-related (c), muscle contraction-related (d), and oxidative stress-related (e) pathways across TM cell populations. Pathways are shown as fold enrichment, with upregulated pathways (increased in GTM) indicated in red and downregulated pathways (decreased in GTM) in blue.

MAPK-related pathways were significantly enriched in GTM (Fig. 5), indicating cluster-specific activation of MAPK signaling across TM populations. TM-JCT and TM-Myo cells showed enrichment of pathways associated with the p38 MAPK and ERK1/2 cascades, including their positive regulation, while TM-Fib cells exhibited enrichment of the p38 MAPK cascade and JNK kinase binding. In contrast, TM-BeamB cells showed downregulation of ERK1/2-related pathways, whereas TM-Uveal cells displayed enrichment of positive regulation of the p38 MAPK cascade (Fig. 8a). Consistent with these pathway-level changes, several MAPK-associated genes showed compartment-specific differential expression (Fig. 8b). In TM-JCT cells, *BMP2*, *GADD45B*, *SPRED3*, *PTGER4*, *AKAP12*, *INHBA*, *ADRA1A*, *ZFP36*, and *CHI3L1* were upregulated. TM-Myo cells showed increased expression of stress-responsive genes *ATF3* and *DUSP1*, along with *AKAP12* and *INHBA*, while TM-Fib cells exhibited elevated *MAD2L2*, *GADD45B*, *INHBA*, and *DUSP1*. In contrast, receptor and signaling genes, including *FGFR2*, *FGFR3*, *FGFR4*, *NRG1*, *EPHB1*, *PKHD1*, and *SLC30A10*, were downregulated in TM-BeamB cells, whereas *GADD45B* was also increased in TM-Uveal cells. These findings indicate cluster-specific modulation of MAPK signaling across TM populations in GTM.

To assess whether MAPK alterations were associated with proliferative changes, cell cycle analysis was performed across TM populations (Supplementary Fig. 13a–f). TM cells were predominantly in the G1 phase across all clusters, with low G2/M and S phase scores and minimal differences between NTM and GTM, indicating a largely quiescent state. However, TM-BeamA and TM-BeamB populations showed significant condition-associated shifts in cell cycle distribution, with TM-BeamA cells primarily altered in G1 phase and TM-BeamB cells in both G1 and S phases. These changes, together with differences in the proportions of TM-BeamA and TM-BeamB cells in GTM, suggest alterations in cellular state rather than increased proliferation.

### Endoplasmic Reticulum (ER)

ER-related pathways were differentially enriched across TM cell populations in GTM (Fig. 5). TM-JCT cells showed downregulation of ER-to-Golgi vesicle-mediated transport, whereas TM-Fib and TM-Uveal cells exhibited enrichment of pathways associated with regulation and negative regulation of ER stress-induced intrinsic apoptotic signaling (Fig. 8c). Consistent with these changes, ER-associated genes displayed compartment-specific differential expression (Fig. 8b). The stress-response gene *HSPA1A* was upregulated in TM-BeamB, TM-Fib, and TM-Uveal cells, while the ER trafficking gene *SORL1* was downregulated in TM-BeamA, TM-BeamB, and TM-JCT cells. These findings indicate compartment-specific modulation of ER stress responses and protein trafficking across TM populations in GTM.

### Integrated stress and contractility pathways underlying TM remodeling in GTM

Multiple biologically relevant processes, including muscle contraction, oxidative stress, shear stress response, wound healing, and outflow tract morphogenesis, were differentially enriched across TM populations in GTM. Muscle contraction and oxidative stress are emphasized due to their close links to calcium signaling, ECM contractility, and metal ion dysregulation, with the remaining pathways detailed in the Supplementary Information.

### Muscle contraction-related pathways

Muscle contraction-related pathways were examined due to their role in the pulsatile biomechanical behavior of the TM. These pathways showed cell-type-specific enrichment across TM populations in GTM (Fig. 8b & d). TM-BeamA and TM-CS cells exhibited downregulation of contractile pathways, including muscle contraction and contractile actin filament bundle assembly, indicating reduced contractile activity. In contrast, TM-BeamB cells showed enrichment of smooth muscle contraction pathways, although contractile actin filament bundle assembly was downregulated. TM-JCT cells displayed mixed regulation, with enrichment of smooth muscle-related pathways alongside downregulation of striated, cardiac, and general muscle contraction pathways. TM-Uveal cells similarly showed downregulation of muscle contraction pathways (Fig. 8d). Consistent with these pathway changes, genes associated with ion channel activity, cytoskeletal organization, and contractile signaling showed compartment-specific DEs (Fig. 8b). The calcium channel gene *CACNA1G* was significantly downregulated in TM-JCT cells, while potassium channel genes (*KCNIP1, KCNJ12*, and *KCNJ3*) were reduced expression across TM populations, particularly in TM-BeamA cells. Structural and contractile genes (*DNM2, TTN*, and *MYL5)* were also downregulated in TM-CS, TM-JCT, TM-Fib, and TM-BeamA cells. Genes involved in cellular signaling and regulatory processes further exhibited DE across TM populations. *ADRA1A* was upregulated in TM-JCT cells, whereas *ARHGAP28* was reduced in TM-BeamA and TM-BeamB cells. The signaling kinase *MAP2K6* and additional signaling-related genes, including *RAPGEF3* and *TMEFF2*, were downregulated across multiple TM populations, while cytoskeletal and vesicle-associated genes such as *STAC2* and *SYNPO* were decreased in TM-BeamB cells. In contrast, several regulatory and ECM-associated genes were upregulated in specific TM compartments, including *PTGER3* in TM-BeamB cells and *PTGER4*, *PTGS2*, *SULF1*, *SDC4*, and *PHLDB2* in TM-JCT cells (Fig. 8b). Together, this analysis indicates cell-type-specific regulation of contractile pathways contributing to TM dysfunction in glaucoma.

### Oxidative stress-related pathways

Oxidative stress-related pathways were enriched across TM cell populations in GTM. TM-BeamA cells showed enrichment of oxidative stress, reactive oxygen species (ROS) metabolic pathways, while TM-BeamB cells exhibited enrichment of cellular response to oxidative stress (Fig. 8e). Consistent with these pathway alterations, several DEGs (*AGT, HGF, HP, HSPA1B*, *SIRPA*) associated with oxidative stress were upregulated in TM-BeamA and TM-BeamB cells. In contrast, *HSPA1A* was upregulated in TM-BeamB, TM-Fib, and TM-Uveal cells, while *SLC1A1* showed significant upregulation in TM-Myo cells (Fig. 8b). These observations indicate activation of oxidative stress-responsive pathways across TM populations in GTM.

## Discussion

Here, we performed snRNA-seq of normal and glaucomatous human TM to define cell-type-specific transcriptional alterations associated with glaucoma. We show that glaucomatous TM is characterized by selective remodeling of distinct TM cell populations, including shifts in cellular composition and coordinated changes in pathways related to metal ion homeostasis, inflammation, calcium signaling, autophagy and mitophagy, contractility, and cellular stress responses. Structural TM populations, particularly TM-JCT, TM-BeamB, TM-Fib, and TM-Myo cells, exhibited prominent disease-associated transcriptional signatures, suggesting key contributions to outflow dysfunction. Together, these findings provide a high-resolution transcriptomic atlas linking TM cellular heterogeneity to impaired AH outflow and elevated IOP in glaucoma.

Prior single-cell and spatial transcriptomic studies in healthy human TM and wild-type mouse models have established baseline cellular composition, regional specialization, and gene expression profiles of the outflow pathway^16–23^. In glaucomatous conditions, mechanistic insights have largely been derived from animal models, particularly the *Lmx1b* mutant mouse, which represents a genetic form of glaucoma with limited relevance to the broader spectrum of POAG^15^. Similarly, single-cell studies in glaucomatous non-human primates have identified changes in contractility-related genes and tissue remodeling^24^, but direct transcriptomic characterization of human POAG TM remains largely unexplored. A major gap remains in defining disease-relevant cellular and molecular changes in human glaucomatous TM, as prior studies have either relied on genetic animal models or limited human datasets. Importantly, Tian *et al*. demonstrated that cultured TM cells rapidly lose tissue-specific transcriptomic signatures, highlighting the necessity of profiling intact tissue to capture physiologically relevant and disease-associated states^20^. To address this gap, we generated a high-resolution snRNA-seq atlas of TM from seven glaucomatous and seven healthy donor eyes, comprising over 285,000 nuclei. This approach enables direct, cell-type-resolved comparison between normal and POAG TM, allowing identification of disease-associated cellular states and molecular mechanisms not fully recapitulated in experimental models. Our study therefore provides a clinically relevant framework to define TM dysfunction in POAG and identify potential therapeutic targets.

### Disease-Associated Shifts in TM Cell States

**Figure 9:**
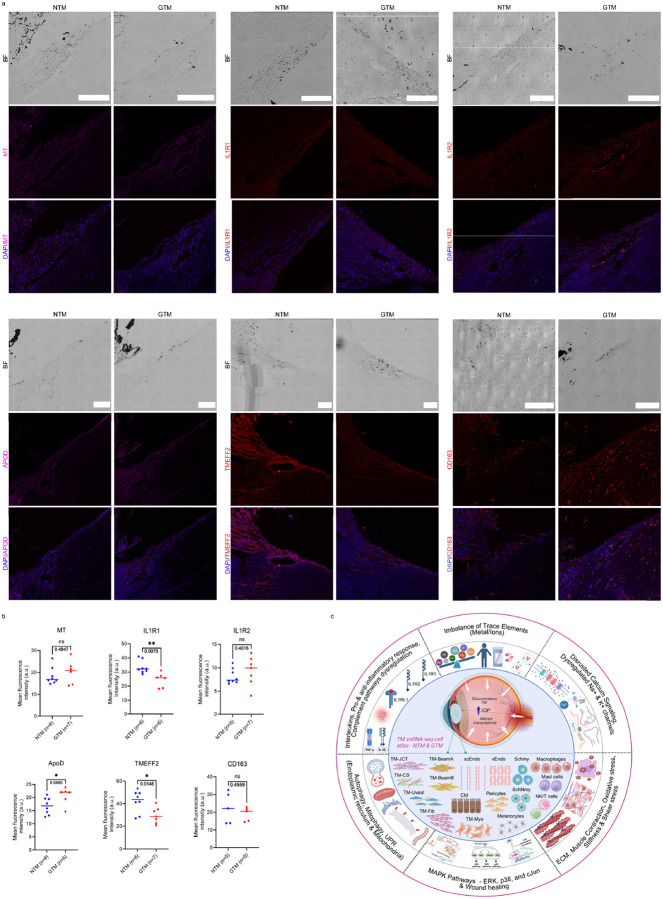
Validation of TM alterations and model of glaucomatous remodeling. a) Representative brightfield (BF) and IHC images of NTM and GTM showing MT, IL1R1, IL1R2, APOD, TMEFF2, and CD163 expression. DAPI (blue) marks nuclei; target proteins are shown in red. Scale bars, 150 μm. b) Quantification of mean staining intensity for indicated markers in NTM and GTM. Data are mean ± SEM.; Pvalues are shown; ns, not significant. Schematic summarizing convergent pathways in glaucoma, including metal ion dysregulation, impaired calcium signaling, inflammatory activation, mitochondrial/proteostatic stress, autophagy, and ECM remodeling, leading to increased contractility, TM stiffening, and impaired AH outflow.

Our snRNA-seq analysis revealed that glaucoma is associated with selective shifts in established TM cell populations rather than the emergence of new cell types. In particular, we observed significant reductions in TM-JCT and TM-BeamB populations, consistent with prior studies showing that TM cellularity declines with age and is further reduced in glaucoma, especially within the JCT region that plays a central role in aqueous humor outflow resistance^25–28^. We also noted trends suggesting a shift toward fibroblast- and myofibroblast-like states, in agreement with earlier work showing that mechanical strain and TGFβ-associated signaling promote fibrotic remodeling and myofibroblast differentiation^29–31^. Consistent with these observations, immunohistochemical analysis demonstrated a significant increase in the TM-Uveal marker, ApoD and a corresponding decrease in the TM-BeamB marker TMEFF2 in GTM samples (Fig. 9a–b). These changes reflect condition-associated transcriptional remodeling within existing TM populations rather than the emergence of novel cell states. In addition, modest increases in immune-associated populations were consistent with reports of macrophage and T-cell infiltration in glaucomatous outflow tissues^32,33^. Schwann cell populations showed reciprocal changes, suggesting altered neural support within the TM, a phenomenon not previously described in GTM. Together, these findings indicate that glaucoma reshapes TM cellular states through coordinated structural, fibrotic, and inflammatory remodeling, providing a transcriptomic basis for impaired outflow and elevated IOP.

### Cell-type-specific remodeling of the TM in Glaucoma

Our analysis reveals that distinct TM cell populations undergo coordinated yet cell-type-specific transcriptional remodeling in glaucoma, converging on shared pathogenic processes that reshape outflow tissue homeostasis. Across TM populations, we observe consistent signatures of ECM remodeling, cytoskeletal reorganization, and activation of stress-response pathways, indicating a transition toward a more fibrotic and mechanically stiffened microenvironment. This is further supported by the upregulation of ECM-modifying enzymes, including *ADAMTS* family members, downregulation of structural regulators such as *CPAMD8*, and increased expression of inflammatory mediators such as *IL1RL1*. Together, these changes suggest coordinated matrix remodeling, compromised tissue integrity, and activation of inflammatory signaling pathways, consistent with previous reports in glaucomatous TM and related fibrotic tissues^34–40^.

TM-JCT and beam-associated TM populations exhibit prominent disruption of intercellular communication, calcium signaling, and fluid and ion transport, changes that are likely to compromise AH outflow. Significant upregulation of *LYPD1* in TM-JCT may indicate disruption of JCT-SC endothelial crosstalk, contributing to increased outflow resistance and tissue stiffening; this aligns with its reported role in suppressing endothelial network formation and its association with fibrotic severity^41,42^. Downregulation of *CACNA1G* indicates reduced T-type calcium influx required for TM contractility and cytoskeletal dynamics. Given the central role of calcium signaling in regulating actomyosin contractility and aqueous humor outflow together with reduced expression of the water channel *AQP5 in TM-JCT*, these changes likely compromise coordinated contractile and hydrodynamic responses required for IOP homeostasis. While other aquaporins have been examined in glaucomatous eyes^43–45^, downregulation of *AQP5* in the human TM has not previously been reported, suggesting a novel mechanism by which altered water transport may contribute to increased outflow resistance; its reduced expression in TM-JCT may further compromise coordinated hydrodynamic responses required for IOP homeostasis. In parallel, attenuation of receptor-mediated signaling, ciliary function, and mechanosensory pathways were observed across TM populations, suggesting impaired sensing of pressure- and flow-dependent cues. These pathways play key roles in mechanosensation and fluid flow regulation^46–52^.

A prominent feature across TM cell types in our analysis was activation of stress-adaptive, lysosomal, and detoxification pathways, accompanied by impaired cytoskeletal dynamics and cellular clearance. TM-CS and TM-Myo populations exhibited reduced phagocytic and macropinocytic capacity alongside increased lysosomal activity, consistent with reports of impaired phagocytosis and debris clearance in glaucomatous TM cells^36^, as well as studies linking macropinocytosis and endocytic dysfunction to altered membrane dynamics and cellular homeostasis^53–55^. Suppression of macropinocytosis in our dataset may further reduce membrane plasticity, promote actin stress fiber accumulation, and potentially altering cellular contractility, in line with established roles of Rho/ROCK-dependent signaling in cytoskeletal organization and fibrotic remodeling^29–31,56–59^. We observed a concurrent shift from clathrin-mediated endocytosis toward lysosome-associated vesicular pathways, supporting enhanced intracellular degradation and stress adaptation, consistent with studies of endocytic pathway remodeling^54^. Consistently, our pathway analysis demonstrated downregulation of phagocytic cup formation, podosome assembly, and cell trailing edge dynamics, reflecting impaired actin-mediated force generation and adhesion turnover, as described in studies of cytoskeletal and focal adhesion remodeling^60–62^. Reduced expression of *DNM2* in our analysis further supports these changes, consistent with its role in actin remodeling and phagocytic processes^63,64^. Enrichment of copper-responsive detoxification pathways indicates chronic oxidative and mechanical stress in GTM, aligning with evidence linking trace metal ion responses to cellular stress adaptation and tissue dysfunction^65^, and may contribute to stabilization of actin stress fibers and reduced membrane turnover, collectively altering cellular contractility.

Calcium signaling emerges as a broadly disrupted axis across TM populations in our analysis. Downregulation of CACNA1G, encoding the T-type calcium channel Cav3.1, indicates disrupted calcium influx in TM-JCT cells; consistent with its role in regulating low-threshold calcium entry and channel availability^66,67^, and with reports of calcium dysregulation in glaucomatous TM cells^68^. In TM-Fib cells, downregulation of CATSPER-associated components suggests a deficit in calcium entry, consistent with the role of CatSper channels in mediating calcium influx^69–71^. CatSper channels are activated by progesterone and prostaglandins^69,72^, and prostaglandin signaling regulates TM physiology and uveoscleral outflow, including in response to glaucoma therapeutics such as latanoprost and travoprost^73–75^ supporting a functional link between these pathways in TM. Although classically associated with sperm-specific calcium influx, their detection here suggests a context-dependent calcium regulatory pathway in TM that warrants further validation. More broadly, attenuation of ligand-gated calcium influx, mechanosensitive ion channels, and ciliary-associated signaling suggests impaired calcium-dependent mechanotransduction across TM populations. Mechanosensitive channels such as TRPV4 mediate Ca^2+^ entry in response to fluid shear stress and regulate aqueous humor outflow and intraocular pressure, and their dysfunction has been shown to impair TM function and elevate IOP in glaucoma^76^. TRPV4-dependent calcium entry may also engage inflammatory and stress-response signaling in TM cells, as osmotic imbalance-induced calcium elevations can contribute to cellular dysfunction and downstream inflammatory processes^77^. Collectively, these findings indicate concurrent disruption of calcium signaling and activation of inflammatory pathways in TM cells, consistent with a coordinated stress-adaptive state underlying glaucomatous dysfunction. Disruption of this axis likely contributes to altered cytoskeletal organization and contractile tone in TM cells, consistent with its established role in outflow regulation^68^.

Together, these findings indicate that glaucoma is associated with coordinated, cell-type-specific reprogramming of TM cells, integrating matrix remodeling, impaired mechanotransduction, disrupted calcium signaling, altered fluid transport, and chronic stress adaptation. This remodeling promotes a shift toward fibroblast- and myofibroblast-like states with altered contractile properties and reduced tissue compliance, ultimately contributing to increased AH outflow resistance and elevated IOP.

### Convergent cellular stress pathways in GTM

Despite marked transcriptional heterogeneity across TM cell populations, our analysis reveals convergence on a core set of stress-associated pathways in glaucoma, with metal ion dysregulation emerging as a prominent feature. Multiple TM populations showed coordinated upregulation of *MT* genes *(MT1A, MT1X, MT1M, MT2A*), consistent with enhanced cellular responses to metal ion and oxidative stress^78–81^, alongside alterations in ion transport systems^82^. These changes likely reflect adaptive mechanisms to buffer excess metal ions and maintain redox balance, in line with established roles of *MT* in metal detoxification and oxidative stress regulation^80,81^. IHC analysis showed increased MT staining in GTM compared with NTM, although this difference did not reach statistical significance (Fig. 9a–b). Concurrent remodeling of ion transport pathways, including SLC transporters and potassium channels, suggests disruption of ionic homeostasis and membrane potential regulation. Notably, downregulation of calcium-activated potassium channel activity in TM-JCT cells (Supplementary Fig. 11b) indicates impaired coupling between calcium signaling and membrane excitability, processes known to influence cytoskeletal dynamics and cellular contractility. Collectively, these alterations may contribute to changes in TM biomechanics and increased outflow resistance in glaucoma.

Inflammatory signaling represents a major axis of convergence in glaucomatous TM. Interleukin-associated pathways are broadly enriched across TM populations, accompanied by increased immune cell presence, indicating a chronically activated yet regulated inflammatory environment. Consistent with the complementary roles of *IL1R1* and *IL1R2*, IL1R2 was broadly expressed and modestly increased at the protein level in GTM, whereas IL1R1 protein levels were reduced significantly (Fig. 9a,b), suggesting attenuation of IL-1 signaling via IL1R2-mediated decoy activity. IL1R2 exists in membrane-bound and soluble forms that sequester IL-1 ligands^83,84^, and is inducible by dexamethasone, glucocorticoids and prostaglandins^83,85,86^, indicating that its expression may reflect both therapeutic exposure and activation of anti-inflammatory pathways. In parallel, enrichment of *IL1RL1* across TM populations supports activation of the IL-33/ST2 axis, suggesting coordinated regulation of inflammatory signaling through integration of IL-33-mediated responses with compensatory attenuation of IL-1 activity^87,88^. Concordantly, immune cell populations, including macrophages, mast cells, and T/NK cells, were moderately enriched in GTM, suggesting that TM-intrinsic inflammatory programs promote immune cell recruitment and retention. Consistent with these observations, IHC confirmed CD163^+^ macrophage localization within the TM and around SC in both NTM and GTM, in agreement with prior studies^33,89^, while emerging evidence links altered macrophage activity to dysfunction of conventional outflow tissues^32,90^. Collectively, these findings indicate coexistence of pro- and anti-inflammatory programs within TM, consistent with prior studies, and support a role for neuroinflammatory remodeling in impaired AH outflow^34,82,90–92^.

In parallel, calcium signaling is broadly suppressed across TM cell types, including voltage-, ligand-, and intracellular calcium transport pathways. Given the central role of calcium in regulating TM contractility and mechanotransduction^68,93^, these changes are expected to impair the ability of TM cells to respond to pressure- and flow-dependent cues. This is further reinforced by activation of Rho GTPase and *TGF-β*-associated pathways, promoting a shift toward a more contractile and fibrotic phenotype^94^.

A unifying feature across TM populations in our study is activation of stress-adaptive pathways, including MAPK signaling, ER stress, and autophagy/mitophagy. These responses likely support proteostasis and mitochondrial function under chronic stress, consistent with established roles of these pathways in cellular stress adaptation^95–102^, but may become maladaptive over time and contribute to impaired TM function. Our findings are consistent with our prior studies and those of others, demonstrating that proteostasis and associated cellular processes, such as the unfolded protein response and autophagy, play a role in TM dysfunction in glaucoma^96,98–100,103^

Despite cell-type-specific transcriptional changes, glaucomatous TM converges on shared pathways involving metal ion dysregulation, inflammation, calcium signaling defects, and proteostatic stress. This coordinated reprogramming provides a mechanistic framework for TM stiffening, impaired mechanotransduction, and increased outflow resistance, ultimately leading to elevated IOP.

## Conclusion

Our single-nucleus transcriptomic analysis of human glaucomatous TM reveals coordinated, cell-type-specific remodeling marked by selective loss and reprogramming of key structural populations. These changes converge on shared pathogenic pathways, including dysregulated metal ion homeostasis, chronic inflammation, impaired calcium signaling, and activation of ER stress and autophagy. Together, these alterations drive cytoskeletal remodeling, extracellular matrix changes, and reduced mechanosensory function, promoting a fibrotic, stiffened TM with impaired AH outflow. This study provides a unified, clinically relevant framework linking cellular reprogramming to elevated IOP and identifies potential pathways for therapeutic intervention.

### Limitations of the study

Our single-nucleus transcriptomic map of glaucomatous TM provides unprecedented insight into cell-type-specific states; however, several limitations should be considered. Inter-donor variability and tissue heterogeneity may obscure subtle transcriptional differences, and increasing sample size in future studies will help further refine disease-associated transcriptional changes. In addition, many glaucoma donors were treated with IOP-lowering medications prior to tissue collection, which may influence specific molecular pathways and transcriptional states within TM cells. Age-related factors also represent an important limitation, as both normal and glaucomatous donor tissues were obtained from aged individuals, making it difficult to distinguish disease-specific alterations from aging-associated changes. Furthermore, because tissues were collected at advanced stages of disease, early molecular events driving TM dysfunction and ocular hypertension cannot be fully inferred from this dataset. Transcriptomic data alone cannot confirm protein expression or functional activity, highlighting the need for downstream validation of pathways such as metallothionein-mediated metal detoxification, calcium signaling, and autophagy/mitophagy. Dissociation-based single-nucleus approaches also remove spatial context, limiting direct inference of *in situ* cell-cell interactions within the TM microenvironment. Future studies integrating spatial transcriptomics, proteomic validation, and early-stage experimental models, including mouse models of ocular hypertension, will help further resolve the temporal and mechanistic basis of TM dysfunction in glaucoma.

## Methods

### Processing of TM tissue samples

All research involving human tissue in this study complied with the Declaration of Helsinki and was approved by Biosafety Committees at the University of California, Irvine (UCI). Donor cadaver eye globes were obtained with appropriate informed consent from the Lions World Vision Institute (LWVI, Tampa, Florida), the San Diego Eye Bank (San Diego, California), and the UCI Willed Body Program. Donor eye globes were disinfected by brief immersion in povidone-iodine (betadine) for 2–3 minutes twice, followed by two washes in phosphate-buffered saline (PBS). A circumferential incision was made approximately 1 mm posterior to the limbus using a scalpel, and the globe was opened along this plane with curved scissors, with partial vitreous transection performed when necessary. The anterior and posterior segments were separated, and the lens, iris, and ciliary body were carefully removed from the anterior segment. Residual iris pigment, ciliary muscle fibers, and tissue debris were gently cleared using the edge of a sterile blade, followed by a brief rinse in PBS. The TM was then isolated by blunt dissection, gently lifted with fine forceps, and teased away as a continuous circumferential strand spanning Schwalbe’s line to the scleral spur, including the inner wall adjacent to the juxtacanalicular tissue, as previously described^22^ (Supplementary Fig. 1). TM tissue from 14 donor eyes (7 normal and 7 glaucomatous) was immediately flash-frozen in liquid nitrogen for nuclei isolation and snRNA-seq. Donor demographics, including age, sex, ethnicity, and relevant ocular pathology, are summarized in Supplementary Table 1 (Table S1).

### Nuclei isolation and snRNA-seq on the 10X Genomics platform

Frozen human TM tissue was retrieved directly from liquid nitrogen and processed without prior thawing. All required buffers and solutions were prepared in advance according to the manufacturer’s instructions. Nuclei were isolated using the Chromium Nuclei Isolation Kit (10x Genomics) following the Sample Preparation User Guide (CG000505, Rev. A). During the isolation procedure, nuclei loss was monitored by mixing an aliquot of the supernatant with trypan blue and examining the sample under a light microscope at each step. The final isolated nuclei were imaged at 20x and 40x magnification to assess nuclear integrity and overall quality. Nuclei concentration and viability were determined using acridine orange/propidium iodide (AO/PI) staining.

Library preparation was performed using the 10x Genomics Chromium instrument with the 3′ Gene Expression v4 assay (CG000731, Rev. B), targeting ~ 20,000 nuclei per sample. All 14 samples were uniquely indexed using 10 bp i7 and i5 indices, enabling multiplexing without cross-sample contamination. Sequencing was performed on an Illumina NovaSeq XPlus to generate a total of ~ 1,000 million reads across all samples. The sequencing configuration consisted of 28 bp for Read 1, 91 bp for Read 2, and 10 bp each for Index 1 (i7) and Index 2 (i5). Read 1 captures the 10x cell barcode and Unique Molecular Identifier (UMI), which together identify individual nuclei and distinguish unique transcripts, while Read 2 sequences the cDNA corresponding to the RNA transcript for gene expression analysis. FASTQ files were received from the UCI Genomics and Technology Hub Core Facility and subsequently processed and analyzed using Cell Ranger as described below, corresponding to an average of ~ 50,000 reads per nucleus.

#### snRNA Data Processing:

Raw single-nuclei RNA sequencing data were processed using Cell Ranger(v9.0.1)^104^, aligning transcripts to the Homo sapiens GRCh38 (2024-A) reference genome. Samples exhibiting low sequencing depth or markedly unbalanced cell recovery compared to other replicates were excluded to ensure consistency in downstream analyses. Quality control metrics from Cell Ranger reports were extracted and summarized in Supplementary Table 1. Low-quality cells were identified using the following criteria: (1) expression of fewer than 200 or more than 4,000 unique genes per cell; (2) total UMI counts per cell below 500; (3) mitochondrial transcripts accounting for more than 10% of total expression; (4) log-transformed ratio of detected genes per UMI below 0.85; and (5) simulated doublet scores below 0.25, as estimated by the Scrublet (v0.2.3)^105^ simulation-based doublet detection algorithm. These low-quality cells were excluded from all subsequent analyses.

### Cell Clustering and Annotation

Filtered gene expression data were normalized using the SCTransform implemented in Seurat (v5.3.0)^106^ pipeline, with mitochondrial gene content regressed out. A total of 5,000 highly variable genes were selected using the VST method, and principal component analysis (PCA) was performed using the top 30 principal components. Batch correction and data integration were conducted using Harmony (v1.2.3)^107^. The Harmony-corrected embeddings were used for UMAP visualization, nearest-neighbor graph construction, and clustering. Cell clusters were identified using a shared nearest neighbor modularity optimization algorithm. Clustering resolution was set to 0.85 to ensure detection of sufficient cell populations. Marker genes were identified using COSG (v0.9.0)^108^, and cell subclusters were annotated by comparing these markers with established cell type markers in the publications.

### Differentiation Expression Analysis

DE analysis was performed using a pseudo-bulk strategy. Raw transcript counts were aggregated by cell type and biological replicate to generate pseudo-bulk expression profiles. To minimize batch effects, the expression matrix was adjusted using a negative binomial regression model from the sva (v3.54.0) package^109^. Gender was included as a covariate in the DE model. Age was excluded due to sample collection limitations, as most samples were from older individuals, resulting in an imbalance for both age and glaucoma status. Pseudo-bulk DE analysis was conducted using DESeq2 (v1.46.0)^110^. Genes were considered significantly differentially expressed if the adjusted p-value was less than 0.05 and the absolute log_2_ fold change exceeded 1.

### Pathway Enrichment Analysis

Gene Ontology (GO) enrichment analysis was performed using clusterProfiler (v4.14.6)^111^ with the org.Hs.eg.db (v3.20.0)^112^ database. For each cell type, DEGs were stratified into upregulated and downregulated groups, and enrichment analysis was conducted separately. Pathways with p-values less than 0.05 were considered significant. For significant GO biological process (BP) terms, hierarchical relationships were categorized using OmnipathR (v3.14.0)^113^. When parent and descendant terms represented overlapping biological concepts, descendant terms were removed to reduce redundancy and retain representative pathways. Pathway categories are summarized by a manual check and keyword searching.

### Cell Cycle Scoring

Cell cycle phase assignment was performed using Seurat (v5.3.0)^106^. Cell cycle marker genes were obtained from the built-in reference. For each cell, S and G2/M scores were calculated using the CellCycleScoring function, which computes the average expression of these marker genes after intersecting them with the dataset’s gene matrix. Cells were categorized into G1, S, or G2/M phases based on the relative enrichment of these scores.

#### Visualization:

All visualizations were generated in R (v4.4.2). UMAP embeddings and gene expression scatter plots (Fig. 1; Supplementary Figs. 2, 3, 5, 6) were produced using scCustomize (v3.2.4)^114^. Volcano plots (Figs. 3–4; Supplementary Figs. 7–9) were generated with EnhancedVolcano (v1.24.0)^115^. Lollipop and MA plots were generated using ggplot2 (v4.0.2)^116^. Pathway heatmaps were created using Complex Heatmap (v2.22.0)^117^. For Figs. 6–8, heatmaps were generated using DE’s log fold change and p-value. Statistical significance was defined as follows: ns, not significant; *p < 0.05; **p < 0.01; ***p < 0.001. If unspecified, Wilcoxon tests were used for statistical comparisons.

## Supplementary Material

This is a list of supplementary files associated with this preprint. Click to download.

- SupplementaryTable4Listofmarkergenesandcitations.docx
- SupplementaryTable3Cellcounttable.xlsx
- SupplementaryTable7Listofantibodiesinthisstudy.xlsx
- 2SupplementaryInformationforTranscriptomicAtlasofHumanTrabecularMeshworkUncovers04262026.docx
- SuppFig12.pdf
- SupplementaryTable1Donordemographicdetails.xlsx
- SuppFig3.pdf
- SuppFig9.pdf
- SuppFig1.pdf
- SupplementaryTable2QCsummary.csv
- SuppFig14.pdf
- SupplementaryTable5DEsigresult.xlsx
- SupplementaryTable8Listofgenefullnamesandabbreviations.xlsx
- SuppFig8.pdf
- SuppFig6.pdf
- SupplementaryTable6GOresult.xlsx
- SuppFig4.pdf
- SuppFig10.pdf
- SuppFig11.pdf
- SuppFig7.pdf
- SuppFig2.pdf
- SuppFig13.pdf
- SuppFig5.pdf

## Data Availability

Primary and processed single-cell RNA sequencing data, as well as the source code, will be publicly available following peer review.
